# A phenome-guided drug repositioning through a latent variable model

**DOI:** 10.1186/1471-2105-15-267

**Published:** 2014-08-08

**Authors:** Halil Bisgin, Zhichao Liu, Hong Fang, Reagan Kelly, Xiaowei Xu, Weida Tong

**Affiliations:** Division of Bioinformatics and Biostatistics, National Center for Toxicological Research, US Food and Drug Administration, 3900 NCTR Road, Jefferson, AR 72079 USA; Office of Scientific Coordination, National Center for Toxicological Research, US Food and Drug Administration, 3900 NCTR Road, Jefferson, AR 72079 USA; Department of Information Science, University of Arkansas at Little Rock, 2801 S. University Ave., Little Rock, AR 72204-1099 USA

**Keywords:** Drug repositioning, Bayesian methods, Latent dirichlet allocation, Data mining, Phenome, Side effects, Indications

## Abstract

**Background:**

The phenome represents a distinct set of information in the human population. It has been explored particularly in its relationship with the genome to identify correlations for diseases. The phenome has been also explored for drug repositioning with efforts focusing on the search space for the most similar candidate drugs. For a comprehensive analysis of the phenome, we assumed that all phenotypes (indications and side effects) were inter-connected with a probabilistic distribution and this characteristic may offer an opportunity to identify new therapeutic indications for a given drug. Correspondingly, we employed Latent Dirichlet Allocation (LDA), which introduces latent variables (topics) to govern the phenome distribution.

**Results:**

We developed our model on the phenome information in Side Effect Resource (SIDER). We first developed a LDA model optimized based on its recovery potential through perturbing the drug-phenotype matrix for each of the drug-indication pairs where each drug-indication relationship was switched to “unknown” one at the time and then recovered based on the remaining drug-phenotype pairs. Of the probabilistically significant pairs, 70% was successfully recovered. Next, we applied the model on the whole phenome to narrow down repositioning candidates and suggest alternative indications. We were able to retrieve approved indications of 6 drugs whose indications were not listed in SIDER. For 908 drugs that were present with their indication information, our model suggested alternative treatment options for further investigations. Several of the suggested new uses can be supported with information from the scientific literature.

**Conclusions:**

The results demonstrated that the phenome can be further analyzed by a generative model, which can discover probabilistic associations between drugs and therapeutic uses. In this regard, LDA serves as an enrichment tool to explore new uses of existing drugs by narrowing down the search space.

**Electronic supplementary material:**

The online version of this article (doi:10.1186/1471-2105-15-267) contains supplementary material, which is available to authorized users.

## Background

The phenome, which can be defined as the comprehensive collection of phenotypic information [1], has been studied intensively to provide solutions for disease-centric biological problems [2]. Most studies in this area aim to demonstrate the correlation between genome and phenome, where various approaches have indicated the usefulness of the phenome to arrive at conclusions about diseases [3–5]. For example, a global comparison of the human interactome and phenome through network alignment has also shown that genome-phenome associations can explain causalities on a larger scale [6]. These studies demonstrate that the phenome can be mined by appropriate tools to discover new treatment opportunities for diseases.

Drug repositioning often involves a process of using knowledge accumulated about a drug during the *de novo* drug discovery process, clinical trial, and/or post-marketing surveillance to identify new therapeutic purposes other than the originally intended purpose. Examining drugs with known safety profiles and pharmacokinetic properties can lead to new therapeutic indications more quickly and with less risk. The history of successful drug repositioning is comprised mostly of serendipitous findings, such as alternative indications for sildenafil and thalidomide, but a more systematic approach is advocated to explore the full benefit of this approach. Interest in drug repositioning is increasing and has attracted researchers from academia, government, and industry, many of whom have developed *in silico* solutions to assist repositioning research. These *in silico* approaches demonstrate the potential of systematic study to improve drug repositioning efforts. In general, the reported studies can be classified as either disease-centric or drug-centric approaches [7]. Most of them used molecular, genomic, or phenotypic data [8–14].

As an example of a molecular study for drug repositioning, Keiser *et al.* measured the chemical similarities of drugs consisting of both US Food and Drug Administration (FDA)-approved and investigational drugs and linked the results to drug targets. They reported thousands of potential drug-target associations and experimentally validated 23 of them that may add alternative therapeutic options for diseases [10]. Using genomic data, Iorio et al. assessed drug similarity based on drug-elicited gene expression in cell lines with a network analysis approach. Their work suggested that Fasudil would be effective in the treatment of autophagy, which is a major process in cancer, and this was confirmed experimentally [9]. In a separate study with genomic data, Sirota et al. compared the gene expression profiling elicited by drugs and that profiled for diseases. They considered a drug effective for a disease if the expression profiles reversely matched. A supporting animal study verified that citemedine could be effective for lung cancer [15]. In a follow up study using the same approach, they reported that anticonvulsant topiramate was effective in the treatment of Inflammatory Bowel Disease (IBD) [16].

On the other hand, the use of the phenome to identify new therapeutic treatments has also been explored in the research community. For instance, Campillos *et al.* hypothesized that drugs having common side effects can also treat the same disease and examined 20 drug-drug pairs, of which nine were experimentally verified for alternative therapeutic uses [17]. Yang *et al.* also studied side effects to assess their associations with diseases through statistical tests [13]. They further focused on the drugs that showed a particular side effect but was not mentioned with the strongly associated indication.

Current *in silico* methodologies in drug repositioning, including phenome-based techniques [17, 18], mostly rely on drug-drug similarity measurements which can lead to guilt-by-association [12]. In other words, the search space is often restricted to the most similar drug without taking full advantage of the information embedded in the entire dataset. We proposed that the phenome should be explored with a probabilistic generative model for a comprehensive analysis. Such an analysis may reveal further links between drugs and diseases.

One important consideration for use of the phenome data is its quality. Although a drug’s phenome (i.e., its side effects and indications) should be consistent within a specific population, the information recorded in some sources, may still remain partial. The missing information could be due to the lack of studies and resources to comprehensively and accurately record all the possible side effects and indications of a drug. Given the challenges to gather a reliable phenome collection, SIDER (Side Effects Resource) [19] stands as a well-credited data source with a room to exploit partial knowledge. In other words, some of alternative indications of a drug that are not yet captured in the existing phenome database are ready for discovery, which is the objective of this study.

This study is built on two premises: (1) the allocation of phenotypes in a phenome arising through both on-target and off-target methods follow a probabilistic distribution in which the therapeutic indications of drugs are embedded and (2) the inherent probabilistic relationship of a drug’s phenome is insensitive to the incompletion of the phenome source. Thus, a probabilistic graph model can be built using the partial phenome and used to identify repositioning opportunities.

To build probabilistic associations between existing drugs and possible indications, we used a Bayesian model, Latent Dirichlet Allocation (LDA), which was primarily developed for topic modeling of text documents. With its probabilistic nature, LDA can link documents and words through latent variables known as topics. LDA achieves such relations by manipulating words, which are the only observed variables, in a Bayesian setting where documents are assumed to be a weighted mixture of multiple topics [20]. By analogy, we have extended this procedure to drugs and phenotypes since phenotypes are observable outcomes of drugs after they hit intended/unintended targets. In other words, this mode of action (MOA) mimics like a latent process where therapeutic effects occur along with side effects due to unknown cellular mechanisms. Since we cannot holistically observe the mechanism causing both effects, we may rely on hidden variables that play a role in the observed outcomes. Therefore, phenotypes are said to be probabilistically distributed across drugs by hidden variables and the same distribution can be utilized to complete missing information. More specifically, these hidden variables (topics) can let us discover the potential links between drugs and phenotypes, specifically indications.

LDA has previously been applied in biological studies such as discovering relationships in PubMed articles, mining relational paths in the biomedical data, and grouping FDA approved drugs based on their therapeutic uses and safety concerns [21–23]. However, its ability to suggest potential associations has yet to be investigated for drug repositioning.

In this study, we applied LDA to the SIDER database to approximate the phenome distribution of drugs. To develop a criterion for suggesting new uses of drugs, we first observed the recovery potential of the model by predicting each “missing” indication from the phenome that was deliberately switched to unknown. In doing so, we took the advantage of not only remaining indication information, but also the whole side effect profile, which is another descriptive feature of a drug. Then, we required a prediction to be both significant and ranked within the top-k positions, where k is equal to the number of known indications for a given drug. We refer to this last characteristic as being within the *indication space (IS)* of a drug. Imposing the same criteria to our initial drug-phenome matrix without any perturbation, we further employed this procedure for drug repurposing. In other words, we attempted to make use of our partial knowledge about side effects and indications to systematically cut down the possible number of drug-indication pairs that can suggest alternative indications. Finally, we both examined several suggested uses by supportive evidence in the literature and captured the original indications of some drugs whose indications were not recorded in SIDER.

## Results

**Figure 1:**
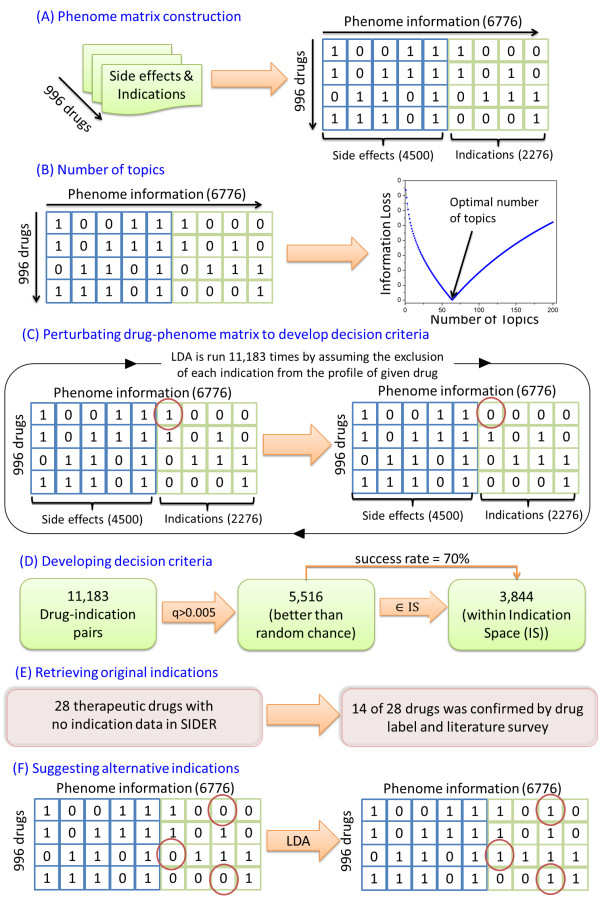
**Overview of the study. A)** Integrating side-effects and indications to complete phenome; **B)** Determining the number of topics by using information loss; **C)** Hiding known indications one by one to see the recovery potential for decision criteria; **D)** Developing the decision criteria considering recovered indications with significance; **E-F)** LDA is applied to the drug-phenome matrix. Observing the decision criteria, real indications were recovered for drugs without indication information and new indications suggested for remaining drugs.

As depicted in Figure 1, this study consists of the following steps: (A) building a drug-phenome matrix with the data profile obtained from SIDER; (B) determining the optimal number of topics by using an information loss criterion to derive a LDA model; (C-D) determining the criteria of the model for application with a procedure that assumed the absence of each indication in a one-by-one fashion; (E-F) applying LDA on the drug-phenome matrix to get probabilities for empty cells, validating the approach by finding known indications mislabeled in SIDER; and predicting new indications for given drugs and validating them through various sources.

### Number of topics

We computed the optimal number of topics for the drug-phenotype matrix by using an information loss criterion (Figure 1B) [18]. When the objective function is minimized, the corresponding number of topics is considered to be the optimal. The information loss reached its minimum when the number of topics hit 64.

### Developing decision criteria

As shown in Figure 1C, a total of 11,183 cases were tested to assess the recovery rate for the masked indications. Specifically, an entry of “1” in the matrix was replaced by “0” one at a time, and then LDA was conducted on the remaining entries and the conditional probability of the indication for the given drug *p*(*I|d*) along with the position of the indication in a ranked list based on probabilities for all indications *p*(*I|d*) were calculated. In order to develop a decision criterion before suggesting alternative indications, we considered the probability of a prediction against chance as well as its location depending on a drug’s IS.

**Figure 2:**
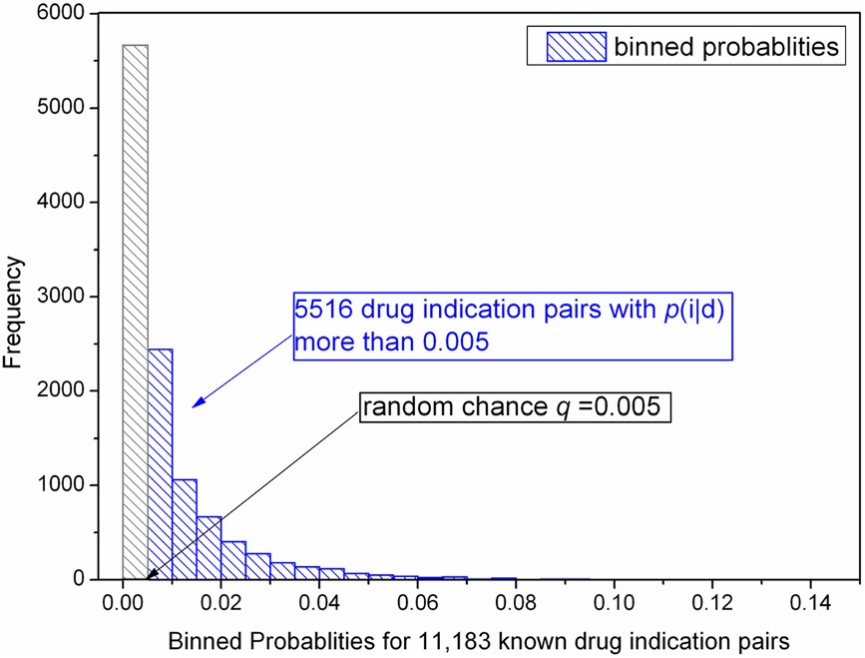
**Binned probabilities for known indications in 11,183 cases.** p(i|d) stands for the probability of the indication in a drug-indication pair. Each bar denotes the number of known pairs falling into the probability intervals. Blue bars show the cases over random chance (q = 0.005), and we have 5,516 drug-indication pairs satisfying this condition.

First, as shown in Figure 1D, we rejected predictions below random chance (0.005), which identified 5,516 cases that correspond to bars above 0.005 in Figure 2. Next, the 5,516 cases were further analyzed against each drug’s IS. In 3,844 out of 5,516 cases, the held indication was recovered correctly to be within the drug’s IS, yielding an average success rate of 70% (*s* = 3,844/5,516) within the portion of pairs that has greater probability than the random chance.

Of note, the individual success rate (*s*) varied on a drug’s IS; the rate was lower in the case of drugs with fewer known indications. For example, we calculated the success rate by incrementally removing the drugs with the lower number of indications. An increasing success rate from 70% to 100% was observed for recovering indications. The results demonstrated that high rates were mostly associated with drugs that have a higher number of indications.

### Retrieving original indications

After developing the criteria, which gave a success rate of 70% for the masked indications, we ran LDA on the drug-phenome matrix in order to discover new links between drugs and indications. This section reports findings for the retrieval of original indications and alternative indications for drug repositioning.

The SIDER database has indication information for most drugs along with side effect profiles. However, 39 of the drugs in the database were not associated with any indications, even though 28 of them had known therapeutic uses. We suspected that these 28 compounds should have multiple indications. Therefore, we employed our model to suggest the missing indication(s). We relied on the same decision criteria, but assumed that the IS = 13, which was the average number of indications in the data set. For each of the 28 drugs, we examined the top 13 indications whose probabilities were higher than the threshold. In order to verify drug-indication pairs, we reviewed the reports from the FDA-approved drug labels, DrugBank [24], and the scientific literature. We confirmed that 14 drugs were shown to be effective for at least one indication suggested by the model. In fact, for 6 of these, the model captured the original approved indications (Table 1).

**Table 1:** **Indications retrieved by the model**

Findings supported by drug labels			
Drug	Indication	Rank	Reference
Thioridazine	Schizophrenia	1	DB00679
Mesoridazine	Schizophrenia	1	DB00933
Bromazepam	Insomnia	4	DB01558
Nitrazepam	Insomnia	3	DB01595
Pipotiazine	Schizophrenia	1	DB01621
Nilotinib	Leukemia	6	DB04868
**Findings supported by literature**			
**Drug**	**Indication**	**Rank**	**Reference**
Apomorphine	Anxiety	6	[25]
Metyrapone	Cancer	6	[26]
Prilocaine	Analgesia	3	[27]
Sevoflurane	Sedation	1	[28]
Remifentanil	Sedation	1	[29]
Methohexital	Muscle relaxation	2	[30]
Isoflurane	Sedation	3	[31]
Disulfiram	Ulcer	1	[32]

### Suggesting alternative indications for drug repositioning

**Figure 3:**
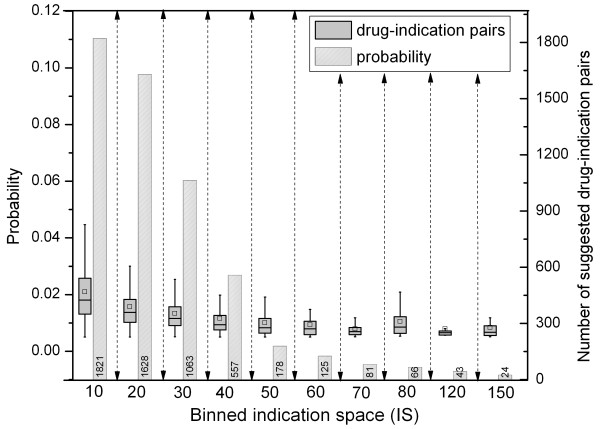
**Binned 5,586 suggested pairs based on IS along with probabilities.** X-axis denotes the number of indications and the bars represent the number of cases (Right-Y-axis) including drugs with that many indications. Box plots summarize the probability distribution (Left-Y-axis) of suggested drug-indication pairs.

Containing 996 drugs with 2,276 indications, SIDER provides a sparse representation of drugs and their indications, which implies that there are many unknown, but possible, drug-indication associations (2,254,830) to be investigated. In that regard, our model worked as a screening technique that might help to reduce the number of candidates for further explorations. As a matter of fact, relying on the established criteria (i.e., beyond random chance and within the indication space of a drug), our model brought up 5,586 potential drug-indication pairs (Additional file 1: Table S1) that correspond to 0.2 % of the total number of possible pairs. For the suggested 5,586 drug indication pairs, the relationship between indication space (IS) and *p*(indication|drug) is illustrated in Figure 3. The figure also provides the number of drug-indication pairs for the drugs, which were grouped based on the IS bins they fall in. These two illustrations show that the number of pairs and probabilities for the pairs with lower IS are greater. This observation implies that for the drugs with smaller IS, the model can suggest alternative indications that are being highly probable and worth to investigate, although it was a challenging task to recover masked indications for the same drugs.

The model offered new treatment options to be investigated for 908 drugs for which two criteria mentioned earlier was satisfied. When the alternative indications were examined, some of the suggestions were confirmed. Table 2 shows a partial list of these confirmations with supportive sources for the new indications that were ranked within the top 15 among 2,276 indications. For instance, amantadine is currently used as medication for influenza A virus and our model suggested alternative uses including the treatment of epilepsy. We conducted a literature search for this suggested treatment option and found that amantadine had been studied and had been beneficial in reducing seizures [33]. Additionally, atazanavir was suggested for use in HIV infection, which is an FDA-approved indication [34] but is not mentioned in SIDER.

**Table 2:** **Verification of suggested uses through literature**

Drug	IS	Indication (source)	Rank of indication (p(i|d))
Atazanavir	6	HIV infection [34]	3 (0.045)
Aripiprazole	15	Dementia [35]	11 (0.033)
Amantadine	18	Epilepsy [33]	7 (0.018)
Itraconazole	15	Meningitis [36]	11 (0.012)
SMS 201-995	36	Migraine [37]	13 (0.015)
Celecoxib	26	Migraine [38]	9 (0.013)
Mefenamic acid	3	Rheumatoid arthritis [39]	3 (0.076)

## Discussion

Experimentally evaluating alternative therapeutic uses of all marketed drugs is time-consuming and labor-intensive. Thus, developing an *in silico* methodology for drug repositioning is a cost-effective way to move the field forward. In this study, we proposed an *in silico* framework which aims to elaborate the entire observed phenome of the available drugs by a probabilistic graphical model. Using LDA, we identified the probabilistic associations of drugs and phenotypes through a latent variable (topic). Under two established decision criteria, we applied the method to infer the probability of new indications for a given drug. The methodology quantifies the nature of a possible relationship that is not previously known. Furthermore, it does not require any *a priori* information because of its unsupervised nature. Another notable advantage of LDA is its flexibility to link a drug with multiple latent variables (topics) through which drugs can be linked to various indications, including those that do not exist in SIDER.

We first established the criteria for the use of LDA on whole phenome by first assuming the absence of each known drug-indication pair and making predictions for that particular indication. For each case, we checked whether the probability (p(*i|d*)) was greater than random chance (p > 0.005) and the indication was ranked within the range of known indications for the given drug (IS). Since *q* = 0.05 is a deterministic threshold for all predictions, it is not allowed to vary with the success ratio that may lead to overfitting. Indeed, its impact remains more unbiased with respect to higher thresholds (Additional file 2: Figure S1). On the other hand, we primarily employed IS as a parametric measure to determine a drug-specific degree of freedom, which enabled us to assess the validity of the model for known pairs and to make a fair comparison. The results show that success rate of the model mostly depends on the number of known indications, with higher rates of success for drugs with many known indications. This is likely because the greater amount of information increased our ability to identify hidden relationships. Nonetheless, the model performed well even on those drugs with fewer indications, with a minimum of 70% success for drugs with at least one known indication.

The capacity of the model to retrieve held-out indications demonstrates its applicability in real-world scenarios. Thus, we made an attempt to retrieve indications for drugs that had been mistakenly omitted in the original dataset from SIDER. Of the 28 drugs with known therapeutic uses in this group, the model was able to identify an indication that had supporting evidence for 14 of them; for 6 of these 14, the indication identified was the approved indication for the drug.

The final goal of the model is to identify novel uses for existing drugs. This model identified 908 drugs from SIDER with new potential indications for further investigations. Similar to the earlier studies, we provided potential pairs that might require an exhaustive search for evidence to verify that the pairs were promising. Therefore, we performed a co-occurrence search in PubMed for the drug-indication pairs and observed that 75% of the pairs were mentioned together in an abstract at least once (Additional file 1: Table S1). Even though co-occurrence cannot be a proof of therapeutic effect alone, it implies the relatedness of many such pairs. Then, since we could not go through the whole list, we made an effort to examine several of the proposed repositioning opportunities to determine if any supporting evidence for the indication existed. We examined the scientific literature, clinical trial data, and approved drug labels to find supportive evidence for the suggested use. The prediction of alternative indications for a drug summarized in Table 2 is based on one Bayesian measure, i.e., the conditional probability of a particular alternative use for a given drug (i.e., p(indication|drug)).

The Bayesian characteristics also allow ranking drugs for queried indications; drugs can be ordered based on the conditional probability p(drug|indication). Using the same strategy, drugs falling within the top k positions (k is the number of drugs that treat a given disease) hold the potential for treatment of a queried disease. One rare disease, Cystic fibrosis, was queried for a candidate drug. Our model suggested ceftazidime for the treatment option, which was indicated earlier in the literature, but was mentioned in neither drug labels nor SIDER [40]. Of note, the p(indication|drug) measure failed to identify the alternative use of ceftazidime for cystic fibrosis, but such an association was uncovered by using the p(drug|indication) measure. The results indicate the utility of both measures for revealing repositioning opportunities.

Among the drugs meeting our decision criteria was thalidomide, a well-known example of a repositioned drug. The model correctly identified the new indication of multiple myeloma for thalidomide. The model, however, is constrained in some respects by our choice of data source. Although SIDER is intended as a comprehensive resource for side effects and indications, not all known indications are included, and these missing indications may affect the performance of the model. For example, buproprion is a well-known example of serendipitous drug repositioning. Initially approved as an anti-depressant (Wellbutrin), clinical observation indicated its potential for smoking cessation. We were unable to identify this new indication using LDA, because this indication does not appear for any drugs in SIDER. This is reasonable that because no drug in the dataset is associated with smoking cessation it is not possible to identify that as an association using only this data set.

Besides the expected uses mentioned above, we analyzed the outcome of the model for off-class uses (off-therapeutic class uses; e.g., using an infection drug to treat obesity). We examined the agreement between the Anatomical Therapeutic Chemical (ATC) Classification System codes of the drugs and the top MeSH (Medical Subject Headings) disease hierarchy for the suggested indications. For 418 drugs with only one ATC code, we checked whether the model offered any indication for off-class use. For 66% of the pairs, we found indications for an additional therapeutic class (Additional file 1: Table S1). For instance, an antineoplastic agent (ATC code: L), thalidomide, was suggested for the treatment of osteoporosis, which is a musculoskeletal disease. This new therapeutic class for thalidomide was supported by earlier studies [41, 42].

We also compared our results with those generated by others. Yang and Agarwal [13] generated alternative indications for 300 drugs based on side effect profiles from SIDER and indication data from PharmGKB [43]. Of the 300 drugs, 269 were also used in our study, and for 145 of them (54%), the two methods agreed on at least one indication. Of the 28 drugs whose indications were missing in SIDER, we found that only 5 were included in the study by Yang and Agarwal. Among these, 4 had at least one new indication consistent with our prediction. In addition, Sirota *et al*. compared gene expression signatures for 100 diseases and 164 drugs and concluded that a reverse correlation between expression changes would help to identify candidate drugs for given diseases [15]. They predicted valproic acid for the treatment of brain tumor and esophagus, lung, and colon cancers. Our model predicted its potential use for tumor and metastases, which has also been supported by an earlier study [44].

Side effects and indications provide a view to the mechanism of a drug, and by using LDA we might be able to use this information to better understand the hidden relationship between a drug, its therapeutic uses, and the side effects it causes. This information can then be used to identify new potential uses for drugs, and our model provides both a probability and rank order to assess these uses. This approach shows significant promise for improving the understanding of identifying new uses for existing drugs as well as drug-topic-side effect relationships that could be used for adverse event prediction. However, the latter task requires more exhaustive procedure and is beyond this study, because the number of perturbations needed is much higher.

Finally, a number of caveats should be noted regarding the present study. The most important limitation lies in the fact that we only have a partial knowledge about the phenome represented by SIDER. While we are perturbing the drug-phenome matrix for recovering indications to observe the potential of partial knowledge, the degree of perturbation remains an open question. This issue might be better addressed by conducting a study on a simulated data set where accuracy can be measured precisely. Secondly, the indication space (IS) proposed in the study may limit the real world application of the methodology, since drug IS is not available. Thirdly, LDA imposes a multinomial distribution on the phenome and it may work better with higher accuracies when the real distribution is close multinomial distribution. Last but not least, our model does not allow sensitivity and specificity measurements due to the unknown nature of the data set.

## Conclusion

In this work, we proposed an *in silico* framework for drug repositioning guided by the phenome that can narrow down candidate indications for given drugs and applied it to SIDER. We treated the phenotypic information of a drug as a fingerprint and assumed it to be generated in a probabilistic fashion. LDA enabled us to retrieve conditional probabilities for new indications for given drugs. Evidence for findings retrieved by the model suggests new opportunities for repositioning, which supports the utility of this model in a systematic repositioning pipeline.

## Methods

### Data source

In the probabilistic graphical model that we employed in this work, we wanted to benefit from all the phenotypic knowledge, i.e., its side effects and indications, associated with the existing drugs. Therefore, we used the most recently updated SIDER data set, which is publicly available at (http://sideeffects.embl.de/) and discussed in depth by Kuhn [19]. SIDER data were collected through Natural Language Processing (NLP) from drug labels and therefore contain some noise. We took all 996 drugs with their phenotypic collections, which consisted of 4500 side effect and 2276 indication terms that exist in MedDRA (Medical Dictionary for Regulatory Activities: http://www.meddra.org/). Then, the input data for LDA became a 996×6776 drug-phenome matrix, where each entry is either 1 or 0 depending on the existence of that particular indication in the drug profile (Figure 1A).

### Latent dirichlet allocation for phenome

**Figure 4:**
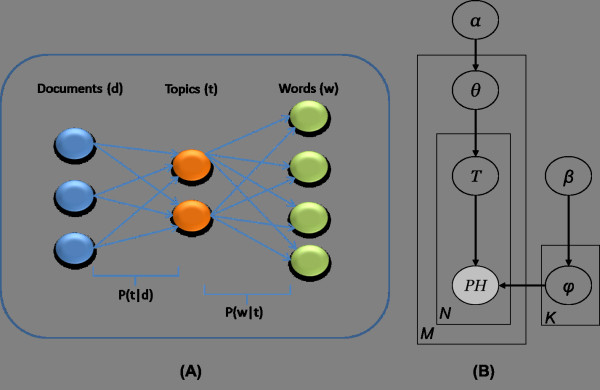
**Relations between variables in LDA model. (A)** Latent variables (topics) are used to construct paths from drugs to phenotypes. P(*t|d*) is the probability that drug *d* is associated with topic *t* while P(*ph|t*) defines the probability of phenotype *ph* associated with topic *t*. **(B)** Graphical representation of LDA for phenome. In this framework, *M* stands for the number of drugs, and *N* is the number of phenotypes.

Latent Dirichlet Allocation (LDA) is a generative model that explains observed data by some latent variables or parameters that give the reason for distribution of the data. For instance, the words in documents are observed and can be explained by unobserved latent variables, i.e., topics, which govern the occurrence and distribution of words within each document. Since LDA assumes each document is a mixture of topics, it associates each document with multiple topics. Similarly, the same setting allows another set of associations between documents and words even for the words that are not observed in that particular document. Figure 4A illustrates this concept with a tri-partite network presentation where each link indicates a conditional probability value and each path defines the strength of association from drugs to phenotypes. In this study, a drug is a “document” while a phenotype of a drug is a “word”. Thus, LDA utilizes its latent components, i.e., topics, as intermediate variables that can discover or suggest such paths even for the unobserved phenotypes (indications as focused in this study) for every drug (i.e., drug).Given the resemblance and the illustration above, we propose an analogous application of LDA for drugs that aims to achieve drug-topic-phenome path. It follows that each phenotype is attributable to one of the drug specific topics, and each drug represents a document, which consists of phenotypes emerging through latent variables, namely, topics. Thus, each drug is linked to multiple topics as shown in Figure 4A.

To achieve the probability value associated with each link, LDA employs a fully Bayesian approach which is shown by the graphical representation in Figure 4B. Each plate notation is used in lieu of links to simplify representation in the presence of multiple variables. For instance, the inner plate indicates there are *N* phenotypes (*PH*) and each of them is linked to multiple topics (*T*) which is equivalent to the right-hand side of the graph in Figure 4A. Furthermore, it carries the information that phenotypes are assumed to be allocated to the drugs with a multinomial distribution. Likewise, the outer plate represents multiple links between *M* drugs (*D*) and *θ*, which is the parameter for a multinomial distribution from which topics are sampled. Finally, the full Bayesian framework is achieved by introducing Dirichlet priors (i.e., *α* and *β*) over the multinomial distributions (i.e., Mult(*θ*), Mult(*φ*)) from which *T* and *PH* are sampled, respectively. The mathematical expression for the joint distribution is given as follows:
1

The ultimate goal of LDA is to compute the posterior distributions of the hidden variables from the above expression for given documents. This inference problem was addressed by Blei *et al*. [20] by employing a variational expectation maximization approach, which provides the distributions, *p*(*T|D*) and *p*(*PH|T*). As *T* being common between two plates and having links to both variables *PH* and drug, it can be utilized to construct paths described in Figure 4A by some algebraic manipulations that will be explained in the following subsection.

Another parameter of our model, which is to be given by the user, is the number of topics, *K*. It can be determined by maximizing the likelihood as proposed by Blei, et al. [20] or by minimizing information loss as proposed by Bisgin *et al*. [18]. We used information loss as the measure to determine the optimal number for *T* in this study (Figure 1B).

### Assessing phenotype probabilities

Above we described a generative model, LDA, which estimates the hidden parameters for the observed phenotypes as well as the conditional probabilities that represent the relationship between hidden (*T*) and observed variables (*PH, D*). Namely, it reveals which latent variables account for the allocation of phenotypes for drugs, similar to words in text documents. Likewise, it also identifies the weighted mixture of the latent variables that constitute a drug’s phenotypic profile. To quantify these relationships, we present the conditional probability distributions that were obtained from the output of the LDA model for further algebraic manipulations:

i. For a given topic *t*, the probability that a phenotype *ph* is associated with topic *t* is denoted as *p(ph|t)*
ii. For a given drug *d*, the probability that *d* is associated with topic *t* is denoted as *p(t|d)*

If we sum the products of (i) & (ii) over *t*, we obtain the probabilities of phenotypes conditioned on drugs:

2The resulting expression refers to the existence probability of a phenotype for a given drug or to the existence potential of the paths illustrated in Figure 4A. The probabilistic values stand for a level of confidence that can be ranked from high to low in order to assess the reliability of any potential link. We expected to have high probability values for the phenotypes already observed in the drugs. However, our goal was to investigate those phenotypes, specifically indications that were not currently associated with given drugs, but yield higher probabilities.

While predicting an indication for drug *d*, we separated indication-specific probabilities (*p*(*I|d*)) from the whole matrix of *p*(*ph|d*). Then, we normalized the probability vector *p*(*I|d*) within itself to obtain updated values and compared the probability of a desired indication (*p*(*i|d*)) to the random chance defined in the following sections.

### Developing decision criteria

In the drug-phenome matrix, we deliberately switched a particular indication of a given drug to unknown by changing the value from “1” to “0” as illustrated in Figure 1C. There were 883 indications which were associated with only one drug. For those indications, we did not alter the corresponding cells because it would remove the indication completely from the dataset. For the remaining 11,183 known drug-indication pairs, we removed and tried to recover each pair in a one-by-one fashion (Figure 1C). For instance, Zidovudine has 13 indications, and one of them is HIV. We removed the Zidovudine-HIV link by switching the corresponding matrix element from 1 to 0. After running the model with the updated matrix, we ranked all the indications in the data set for Zidovudine based on the conditional probability, *p(*indication*|*Zidovudine*).* The goal here was to see the ability of model to locate HIV with a probability value more than random chance and within the top 13 ranked indications, which we referred as the *indication space (IS)* of the drug in an earlier section.

In our framework, we define the random chance, *q*, as the probability of choosing an existing drug-indication pair from the pool of all possible pairs. If *N* is said to be the number of known pairs, which will also be used to develop decision criteria, *q* can be calculated as follows:
3

where | *D* | is the number of drugs, and | *I* | is the number of indications in the data set. Thus,
4

Once we completed all the runs for the 11,183 cases, we counted the number of cases that had a probability value greater than the random chance (*q* = 0.005) and were successfully found within the given drug’s IS. Specifically, as illustrated in Figure 1D, the cases that have probabilities less than 0.005 were first discarded since they were not considered reliable. For the remaining portion, we only considered the trials that were ranked within the IS and the success rate was calculated as below:
5

In summary, this excise resulted in two criteria to assess the reliability of a model’s prediction. That is, in this studied matrix, a new indication of a drug is considered to be valid only if its existence is beyond random chance and its ranking by probability is within the IS of a drug.

### Retrieving and suggesting indications

The above criteria were subsequently used for predicting new indications of drugs by applying LDA to the drug-phenome matrix in Figure 1B. Unlike in the previous section, we did not make any changes in the matrix and attained probabilities for the non-existing links between drugs and indications that correspond to the entries with “0”. Hence, probabilities and rankings for the indications were utilized to retrieve original indications of some drugs that were recorded in SIDER without indication information. Furthermore, the same procedure along with the decision criteria proposed alternative treatment options for the drugs whose indications were mentioned in SIDER. In total, we identified 5,586 potential drug-indication pairs in the matrix that met our two criteria out of the 2,254,830 unknown drug-indication pairs (or 996x2276-(11183 + 883) empty cells). In other words, this practice curtailed the pairs to focus on a list that can promise repositioning opportunities.

## Electronic supplementary material

Additional file 1: Table S1: Suggested pairs were provided along with their probabilities, ranks, off-system use, and literature mining results. (XLSX 504 KB)

Additional file 2: Figure S1: *q* (cut-off) dependency of success ratio. Dashed line indicates our deterministic threshold (random chance). If *q* was varied, success ratio would have changed as illustrated in the curve. (JPEG 42 KB)
