# Supplementary figures and images for: A phenome-guided drug repositioning through a latent variable model
Source: BMC Bioinformatics. 2014 Aug 8;15(1):267. doi: 10.1186/1471-2105-15-267 (PMC4137076; doi:10.1186/1471-2105-15-267)

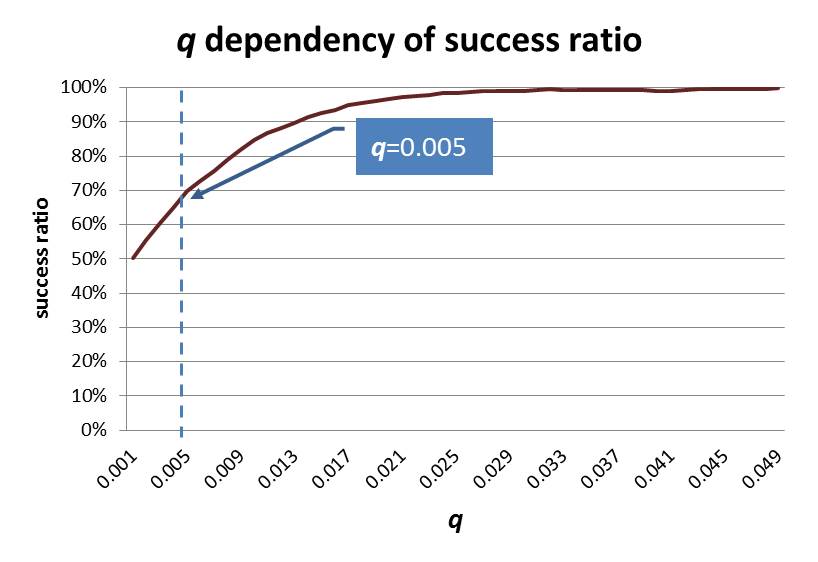

Supplement: Supplementary file 2 — Additional file 2: Figure S1: q (cut-off) dependency of success ratio. Dashed line indicates our deterministic threshold (random chance). If q was varied, success ratio would have changed as illustrated in the curve. (JPEG 42 KB) [file 12859_2014_6538_MOESM2_ESM.jpeg]
